# TRAF7 knockdown induces cellular senescence and synergizes with lomustine to inhibit glioma progression and recurrence

**DOI:** 10.1186/s13046-025-03363-1

**Published:** 2025-04-04

**Authors:** Yu Chen, Tongyu Zhou, Rongrong Zhou, Wen Sun, Yan Li, Qiyi Zhou, Dongcheng Xu, Yuxin Zhao, Peihao Hu, Jingrui Liang, Yumeng Zhang, Bin Zhong, Juncheng Yao, Di Jing

**Affiliations:** 1 Department of Oncology, Xiangya Hospital, Central South University, Changsha, 410008 China; 2 National Clinical Research Center for Geriatric Disorders, Xiangya Hospital, Central South University, Changsha, 410008 China; 3 Department of Global Health and Social Medicine, King’s College London, London, UK; 4 Key Laboratory of Animal Biological Products & Genetic Engineering, Ministry of Agriculture and Rural, Sinopharm Animal Health Corporation Ltd, Wuhan, 430023 China; 5 State Key Laboratory of Novel Vaccines for Emerging Infectious Diseases, China National Biotec Group Company Limited, Beijing, 100024 China; 6 Center of PRaG Therapy, Center for Cancer Diagnosis and Treatment, Laboratory of Cancer Radioimmunotherapy, The Second Affiliated Hospital of Soochow University, Suzhou, 215004 China; 7 Department of Spine Surgery, The Third Xiangya Hospital, Central South University, Changsha, 410008 China; 8 College of Life Science and Technology, Huazhong University of Science and Technology, Wuhan, 430074 China; 9 Department of Neurosurgery, Hunan University of Chinese Medicine Affiliated Yueyang Hospital, Yueyang, 414000 China; 10 Dalian Medical University, Dalian, 116041 China

**Keywords:** Glioma, Recurrence, Lomustine, TRAF7, Cellular senescence, G0/G1 arrest

## Abstract

**Background:**

The progression and recurrence are the fatal prognostic factors in glioma patients. However, the therapeutic role and potential mechanism of TRAF7 in glioma patients remain largely unknown.

**Methods:**

TRAF7 RNA-seq was analysed with the TCGA and CGGA databases between glioma tissues and normal brain tissues. The expression of TRAF7, cellular senescence and cell cycle arrest pathways in glioma tissues and cell lines was detected by real-time quantitative PCR (RT-qPCR), western blotting and immunohistochemistry. The interaction between TRAF7 and KLF4 was determined by Co-immunoprecipitation (Co-IP) assays. The functions of TRAF7 combined with lomustine in glioma were assessed by both *in vitro*, *in vivo* and patient-derived primary and recurrent glioma stem cell (GSC) assays.

**Results:**

High TRAF7 expression is closely associated with a higher recurrence rate and poorer overall survival (OS). *In vitro*, TRAF7 knockdown significantly inhibits glioma cell proliferation, invasion, and migration. RNA-seq analysis revealed that TRAF7 inhibition activates pathways related to cellular senescence and cell cycle arrest. In both *in vitro* and patient-derived GSC assays, the combination of sh-TRAF7 and lomustine enhanced therapeutic efficacy by inducing senescence and G0/G1 cell cycle arrest, surpassing the effects of lomustine or TRAF7 inhibition alone. Mechanistically, TRAF7 interacts with KLF4, and a rescue assay demonstrated that KLF4 overexpression could reverse the effects of TRAF7 depletion on proliferation and cellular senescence. *In vivo*, TRAF7 knockdown combined with lomustine treatment effectively suppressed glioma growth.

**Conclusion:**

TRAF7 could be used as a predictive biomarker and the potential therapeutic target among National Comprehensive Cancer Network (NCCN) treatment guidelines in the progression and recurrence of glioma. Lomustine, regulating cellular senescence and cell cycle could be the priority choice in glioma patients with high-level TRAF7 expression.

## Key points

• High TRAF7 expression is linked to glioma progression and recurrence.

• TRAF7 knockdown inhibits glioma cell proliferation, invasion, and migration via cellular senescence and cell cycle arrest.

• Lomustine may be prioritized for glioma patients with high-level TRAF7 expression.

## Importance of the study

Our study highlights the clinical relevance of TRAF7 as a potential biomarker and therapeutic target in glioma treatment. By demonstrating that TRAF7 knockdown significantly inhibits glioma progression and enhances the efficacy of lomustine, particularly in high-TRAF7 expression subtypes, it opens avenues for more targeted and effective therapies. The use of both public datasets and glioma-bearing mouse models further strengthens the translational potential of these findings. These insights into TRAF7 signaling provide a crucial step toward personalized glioma therapies, potentially improving patient outcomes in progression and recurrence.

## Introduction

Glioma originating from glial or precursor cells represents the most prevalent and aggressive type of primary brain malignancies, constituting 80.9% of central nervous system malignancies [1, 2]. The brain malignant tumor includes high-grade gliomas (HGGs, III-IV grade) and low-grade gliomas (LGGs, I-II grade). HGGs primarily depend on gross total resection, and subsequently adjuvant radio-chemotherapy can reduce recurrence rates and prolong overall survival. However, the five-year survival rate typically falls below 10% [3]. Approximately 52%–62% LGG patients experience a recurrence within 5 years and there exists a high risk of progression to HGG among these recurrent LGG patient, which ultimately results in poor survival prognosis [4, 5]. Biomarkers are important factors associated with glioma prognosis, including IDH mutations, CDKN2a deletion, and TERT promoter mutations [6]. Cellular senescence widely exists in the tumorigenesis of brain cancer, to explore the potential senescence-associated checkpoints is an important clinical problem in the adverse prognosis subtype of glioma based on analysis of public databases.

Cellular senescence refers to a permanent cessation of cell division triggered by multiple stressors, including oncogenic activation and chemotherapeutic agents [7, 8]. Senescent cells retract from the cell cycle through the activation of the p53/p21^CIP1^ and p16^INK4a^/Rb tumor suppressor signaling pathways [9]. Inducing cells into senescence operates as an anti-tumor barrier, and the tumor-suppressive function is further enhanced by senescence-associated secretory phenotype (SASP) in cell-extrinsic environment [10, 11]. Pallavicini et al. have demonstrated that CITK deletion induced p53-dependent senescence to limit proliferation in medulloblastoma [12].

Tumor necrosis factor receptor (TNFR)-related factors (TRAFs) are a class of cytoplasmic mediator proteins, and the majority of TRAFs additionally serve as E3 ubiquitin ligases to initiate downstream signaling processes, resulting in nuclear factor-κB (NF-κB), mitogen-activated protein kinase (MAPK), or interferon-regulatory factor (IRF) activation [13, 14]. The canonical members (TRAF1-6) participate in various activities and play a role in immune and inflammatory -related responses [15, 16]. Therefore, TRAF7 as a non-canonical member of TRAF family has been less studied. Dr. Zhang found that hepatocellular carcinoma (HCC) with TRAF7 high expression was closely related with poor prognosis though repressing cell apoptosis [17]. In the study of meningioma, TRAF7 deficiency activated the RAS/MAPK pathway and promoted the tumor progression [18]. However, the molecular mechanisms of TRAF7 through senescence in glioma remain largely unknown.

Here, we generated a comprehensive analysis of TRAF7 in glioma. First, we explored the connection between TRAF7 expression and clinical prognosis in the TCGA and CGGA datasets, and verified this association in the real-world patient dataset. We discovered that TRAF7 inhibition activates cellular senescence and cell cycle arrest pathways *in vitro*. Furthermore, the combination of TRAF7 inhibition with the second-line drug lomustine, as suggested by the National Comprehensive Cancer Network (NCCN) treatment guidelines, exhibited notable anti-tumor effects both *in vitro* and in patient-derived GSC assays. Mechanistically, TRAF7 interacts with KLF4, and a rescue assay showed that KLF4 overexpression could reverse the effects of TRAF7 depletion on proliferation and cellular senescence. These findings highlight TRAF7 as a prognostic indicator and a potential therapeutic target in precision medicine, suggesting that early administration of lomustine could benefit glioma patients with high TRAF7 expression.

## Materials and methods

### Data acquisition and processing

The datasets of gene expression and clinical details of glioma patients were downloaded and normalized by R package “limma”from TCGA database (https://portal.gdc.cancer.gov/). Altogether 704 cases were included in this research analysis. Similarly, the 693 RNA­seq and associated clinical data were retrieved from the Chinese Glioma Genome Atlas (CGGA) (http://www.cgga.org.cn) and were normalized as a validation set.

### Clinical data collection

One hundred forty-three glioma samples were retrospectively collected in Xiangya hospital, including 76 recurrent samples. The study was approved by the Ethics Committees of Xiangya Hospital (approved number: 2023081128). The detailed clinicopathological characteristics were shown in Table 1.

**Table 1:** Clinicopathological parameters of Glioma in Xiangya Cohort

Clinical pathological indexes		TRAF7 high expression	TRAF7 low expression	*P* value
Age	≥ 45	46	12	< 0.001
	< 45	44	41	
Gender	male	61	32	0.301
	female	29	21	
WHO grade	II	17	45	< 0.001
	III	36	8	
	IV	37	0	
Subtype	Astrocytoma	6	29	< 0.001
	Oligodendroglioma	1	3	
	Ependymoma	2	1	
	Ganglioglioma	0	6	
	Glioblastoma	81	14	
Recurrence	Yes	63	13	< 0.001
	No	27	40	
DFS	≥ 60	35	41	< 0.001
	< 60	55	12	
OS	≥ 85	42	31	< 0.001
	< 85	48	22	
Ki67 positive %	≥ 15	25	3	0.263
	< 15	65	50	
EGFR positive %	≥ 80	53	24	0.280
	< 80	37	29	

### Nomogram model construction

With the multivariate cox regression analysis as a foundation, a predictive nomogram model was created using the 'rms' and 'survival' R packages. This nomogram predicts the 1-year, 3-year, and 5-year overall survival probabilities for glioma patients, with calibration curves produced to validate its accuracy. In addition, decision curve analysis (DCA) was applied to measure the net clinical benefits provided by the nomogram model.

### Cell culture

The human glioma cell lines Hs683 and U251 were sustained in DMEM medium (Gibco, USA), with the addition of 10% fetal bovine serum (FBS) and 1% penicillin/streptomycin at 37℃ under a humidified incubator containing 5% CO2.

### Real-Time quantitative PCR (RT-qPCR)

TRIzol reagent (ET101-01, Transgen biotech, China) was utilized to isolate total RNA. cDNA synthesis was carried out with Thermo Scientific RevertAid RT kits (Thermo Fisher Scientific, Carlsbad, CA) following the manufacturer's instructions. qRT-PCR was carried out with a SYBR Green mix (Thermo Fisher Scientific, Carlsbad, CA) on a ViiA™ 7 RT-PCR system (Applied Biosystems, Carlsbad, CA). The detailed information was shown in Table S1. GAPDH served as the internal control gene.The relative mRNA levels were calculated by the 2-ΔΔCt method.

### Western blotting assay

Total protein extraction was done by RIPA buffer (89900, Thermo Fisher Scientific, Carlsbad, CA) containing a protease inhibitor (P1006, Beyotime, Shanghai, China). Following 10% SDS-PAGE separation, proteins were then transferred onto polyvinylidene fluoride (PVDF) membranes (88518, Thermo Fisher Scientific, Carlsbad, CA). Then, the membrane was treated with primary antibodies at refrigerated temperature for a duration of 12 h. The primary antibodies are as follows: TRAF7 (1:500, AF08297, AiFang biological, China), CDK2 (1:1000, ab32147, Abcam, UK), CDK4 (1:1000, ab108357, Abcam, UK), CCND1 (1:2000, ab134175, Abcam, UK), CCNE1 (1:2000, 11554–1-AP, Proteintech, China), CCNB1 (1:1000, ab215436, Abcam, UK), ATM (1:2000, ab32420, Abcam, UK), P-ATM (1:2000, ab81292, Abcam, UK), ATR (1:1000, #2790, CST, USA), P-ATR (1:1000, #2853, CST, USA), CHK (1:2000, ab235938, Abcam, UK), P-CHK (1:1000, 28805–1-AP, Proteintech, China), γ-H2AX (1:2000, 10856–1-AP, Proteintech, China), P16 (1:1000, 10883–1-AP, Proteintech, China), P53 (1:2000, #2527, CST, USA), P21 (1:1000, #2947, CST, USA), MMP3 (1:2000, 17873–1-AP, Proteintech, China), IL6 (1:2000, 21865–1-AP, Proteintech, China), and β-actin (1:1000, 81115–1-RR, Proteintech, China). The following day, the membranes were incubated for 40 min at room temperature with a horseradish peroxidase (HRP)-conjugated secondary antibody: Goat Anti-Mouse IgG H&L (HRP) (ab6789, Abcam, UK) or Goat Anti-Rabbit IgG H&L (HRP) (ab6721, Abcam, UK). Signal was captured with ChemiDocXRS + System. Image Lab was utilized to perform quantitative protein analysis. Relative expression was quantified by normalizing the intensity of the gene of interest to that of β-actin.

### RNA interference and transfections

The small interfering RNA (siRNA) that targeting TRAF7 was purchased from General Biol (Anhui, China). TRAF7 was silenced using siRNA that targeted the following sequences: sense 5′- GCACACGUUCUGUAGGAGATT −3′ and antisense 5′-UCUCCUACAGAACGUGUGCTT-3′. The sequences of the negative control were as follows: sense 5′-GUAUGACAACAGCCUCAAGTT-3′ and antisense 5′- CUUGAGGCUGUUGUCAUACTT-3′. The cells were seeded in 6-well plates overnight and then transfected separately with TRAF7 siRNA or negative control siRNA using Lipofectamine RNAiMAX Transfection Reagent (Invitrogen, Carlsbad, CA) according to the manufacturer's instructions. After 48 h, the transfected cells were harvested for western blotting and subsequent assays.

### Lentiviral vector construction and stable transfection

Lentiviral constructs were conducted by Shanghai Genechem Co. Ltd. Then sh TRAF7, TRAF7 and KLF4 overexpression lentiviral constructs were transfected into U251 and Hs683 cells using TurboFect (R0531, ThermoFisher, Invitrogen, Carlsbad, CA). Stably transfected cell clones were selected by puromycin (ThermoFisher, Invitrogen, Carlsbad, CA) at 2 μg/mL for two weeks. The efficiency of shTRAF7, TRAF7 and KLF4 overexpression was quantified by real-time PCR and western blotting.

### Cell proliferation assays

Each well of the 96-well plates was seeded with 3,000 cells with 100 μl of complete medium and cultured during the night. At 0, 24, 48, and 72 h, each well received 10 μl of CCK-8 solution (Biosharp, China) and was incubated at 37 °C for 2 h.The absorbance at 450 nm was recorded using a Microplate Reader. Subsequent absorbance readings were used to generate the cell growth curve.

### EdU assay

Each well of the 96-well plates received 3,000 cells in 100 μl of complete growth medium and cells were left to incubate across the night. After 24 h, 20 μM EdU labeling medium was used to grow with cells for 3 h. Following fixation with 4% paraformaldehyde, cells then were incubated with 100 μl of Apollo dye solution (C10310-1, RiboBIO, China) for 30 min. Afterward, the samples were washed with 0.5% Triton X-100 for 10 min. Finally, DAPI was used to stain the cells, and positive cells were subsequently counted using an N2-DMi8 microscope (Leica, Wetzlar, Germany).

### Wound-healing assay

Cells were placed onto 6-well plates and the scratch was made by 10 μL plastic pipette tips. Then each dish was filled with DMEM medium supplemented with 2% FBS. The images of wound healing were photographed via microscopy at the different time points (0 h, 12 h and 24 h). The wound closures were recorded with a N2-DMi8 microscope (Leica, Wetzlar, Germany). Areas covered by migrated cells (%) were quantified by Image J.

### Colony formation assay

Seven hundred glioma cells were seeded per well in 6-well plates and incubated for 14 days. Then after fixed with methanol for 20 min, cells were stained by crystal violet (C0121, Beyotime, Shanghai, China) for 20 min. Colony counts were performed with the aid of Image J software.

### Transwell assay

After twenty-four hours post-transfection, 4 × 10^4^ glioma cells were collected and resuspended in serum-free medium. Cells were subsequently transferred to the upper side of a Transwell insert (Millipore). For the invasion assays, Matrigel was used to coat the Transwell chambers, while 800 μL of 10% FBS medium was added to the lower compartment. In contrast, no Matrigel was applied for the migration assays. After a 48-h cultivation at 37°C, the cells on the inferior side were then rinsed with phosphate-buffered saline, fixed with 4% paraformaldehyde, and then dyed with crystal violet. Photographs were subsequently taken to analyze stained cells using an N2-DMi8 microscope.

### RNA extraction and transcriptome data analysis

Cells treated with TRAF7 siRNA were subjected to RNA extraction using Trizol, and illumina sequencing technology was employed for transcriptome analysis. Gene expression differences were identified through the EdgeR algorithm integrated into the CLC Genomics Workbench (Qiagen, Hilden, Germany). The *p*-value cutoff was set based on the false discovery rate (FDR) to correct for multiple comparisons. An FDR value of ≤ 0.01 was chosen to pinpoint significant gene expression changes during the tripartite bioassay.

### β-Galactosidase staining

β-Galactosidase staining was applied using the Senescence staining kit (C0602, Beyotime, China). Briefly, TRAF7 shRNA group and TRAF7 vector group were treated with lomustine and PBS for 24 h, and subsequently stabilized with Fixative Solution for 15 min at ambient temperature. The cells were treated with β-Galactosidase staining solution at pH 6.0 and incubated at 37°C for 24-h period. Images were captured under bright-field microscopy.

### Cell cycle test

In 6-well plates, 100,000 cells per well were seeded in 2 ml of complete medium and then treated with DMSO or 50 μM lomustine for 12 h. Afterwards, the cells were collected and washed with PBS. Subsequently, DNA staining solution and permeabilization solution (CCS012, Multi Sciences, China) were added and cultured for 30 min at 37°C. The cells were immediately analyzed using a flow cytometer (CytoFLEX, Beckman Coulter, USA).

### Co-Immunoprecipitation (Co-IP) assay

Protein A/G magnetic beads were initially suspended in binding buffer (50 mM Tris, 150 mM NaCl, 0.1%−0.5% Triton X-100 or Tween 20, pH 7.5) and incubated with Flag, HA, or normal IgG antibodies for 1 h at room temperature with end-over-end rotation. After removing the supernatant, the cell lysate was added to each tube and incubated with rotation for 10 min at room temperature. The immunoprecipitated proteins were then released by boiling the samples for 5 min at 95°C in SDS-PAGE sample buffer. The magnetic beads were removed and the samples were loaded onto a 10% SDS-PAGE gel for analysis.

### Patient-derived glioma stem cell (GSCs) sphere formation assay

The tissues were minced into 1–3 mm^3^ pieces and mixture was filtered using a 100 μm cell strainer (258365, NEST, USA), then red blood cell lysis solution was added. Samples were purified by centrifugations in cold PBS. For sphere formation assay, cells were seeded at 100 cells/well in non-serum medium. After 1 week, the diameter of spheres larger than 70 μm were recorded via light microscopy. For sphere formation assay in Matrigel, the collected precipitates were mixed with cold matrigel (Corning, CLS356237, USA) and then deposited in 40 μl droplets into prewarmed 24-well plates (Corning, CAT # 3524, USA). The plates were inverted and incubated for 20 min, and then the glioma stem cell culture medium was added. The medium contained the following components: DMEM/F12 (Gibco, USA), penicillin (Gibco, USA), human insulin (Sigma-Aldrich, USA), N2, B27 serum substitute (Gibco, USA), Glut Amax Supplement (Sigma Aldrich, USA), HEPES (Gibco, USA), recombinant human epidermal growth factor (Sigma-Aldrich, USA), Y27632 (Sigma-Aldrich, USA), recombinant human basic fibroblast growth factor (Sigma-Aldrich, USA).

### Stemness biomarker staining

Cells were transferred onto gelatin-coated glass slides to prepare immunofluorescence staining. Following pre-incubation with 10% normal goat serum and 0.3% Triton X-100 in PBS for 1 h at room temperature, a primary anti-CD133 antibody (ab222782, Abcam, Cambridge, UK) was applied for 18 h at 4°C. Subsequently, the sections were rinsed with PBS, and then incubated with a secondary antibody conjugated to Alexa fluor 488 (ab150077, Abcam, Cambridge, UK) for 1 h at 37 ◦C. Afterwards, the nuclei were stained with DAPI and visualized under a microscope.

### Glioma orthotopic implantation model

Six–eight weeks Balb/c nude mice were fixed on a stereotactic frame (RWD Life Science, China) after anesthetized. Then, 3 µL of U251 luciferase cells (3 × 10^5^) were administered into the right cerebellum of nude mice (1 mm to the right of the midline, 1 mm posterior to the lambdoid suture, and 3.5 mm deep). Nude mice bearing orthotopic U251 brain cancer were distributed randomly into six groups (*n* = 5): Control (TRAF7-Vector), Control (TRAF7-Vector) + lomustine (CCNU), Sh-TRAF7, Sh-TRAF7 + lomustine (CCNU), OE-TRAF7, OE-TRAF7 + lomustine (CCNU). Intracranial glioma cancer cells growth was monitored by bioluminescence imaging (AniView100, Biolight Biotechnology, Guangzhou). After 35 days, nude mice were treated with PBS or CCNU via tail vein injection. According to the clinical dose for the glioma patient treatment (CCNU, 120–130 mg/m^2^), the concentration of CCNU was calculated by the body surface area of mice (20 mg/kg). During the treatment process, mice received intraperitoneal injections of luciferin (Sigma-Aldrich, USA) twice a week to continuously record the alterations of brain tumor by the bioluminescent signal.The mice brain containing tumor tissues were collected to further pathological analysis.

### Hematoxylin–eosin (HE) and Immunohistochemistry (IHC) staining

Ten percent formalin was utilized to fix the whole-brain xenograft tumor tissues, which were then dehydrated, encased in paraffin, and sectioned into 4 µm slices. The sections were dewaxed, hydrated, and underwent antigen retrieval and endogenous peroxidase blocking. The sections were maintained with primary antibodies at 4°C throughout the night: TRAF7 (1:300, 11,780–1-AP, Proteintech, China), Ki67 (1:400, #9449, CST, USA), P53 (1:200, #2527, CST, USA), P21 (1:100, #2947, CST, USA), CCND1 (1:200, ab134175, Abcam, UK), CDK2 (1:100, ab32147, Abcam, UK). Next, the slides were incubated with a secondary antibody (1:1000, ab6721, Abcam, UK) at 25°C for 1 h. After DAB and hematoxylin staining, positive cells were counted using a microscope.

### Multiplex immunofluorescence

FFPE tissue sections were stained using the mIHC Fluorescence kit (RC0086-34RM, RecordBio, Shanghai, China) following the manufacturer’s instructions. The process included deparaffinization with xylene, hydration with gradient ethanol, antigen retrieval with sodium citrate buffer (0.01 M, pH 6.0), removal of endogenous peroxidase with H2O2, blocking with 10% goat serum, and staining with antibodies and TSA-RM. Each cycle of staining began with antigen retrieval and ended with TSA labeling. The antibodies used were: P21 (1:100, #2947, CST, USA) with TYR-520, P53 (1:200, #2527, CST, USA) with TYR-690, CCND1 (1:200, ab134175, Abcam, UK) with TYR-570, Ki67 (1:200, #9449, CST, USA) with TYR-620. Nuclei were stained with DAPI. All slides were scanned and analyzed using the pathology imaging microscope (Pannoramic MIDI II, 3D HISTECH, Hungary).

### Statistical analysis

The data were shown as mean ± SEM and processed through GraphPad Prism 7.0 software. Odds ratios (ORs) and 95% confidence intervals (95% CI) were determined via logistic regression analyses. The Student's t-test was employed to compare two groups, and one-way ANOVA was used for comparing variable groups. Kaplan–Meier survival curves were generated and evaluated using the log-rank test, while predictive factors impacting glioma prognosis were determined via the Cox proportional hazards model. Statistical significance was considered as *p < 0.05, **p < 0.01, and ***p < 0.001.

## Results

### High-expression of TRAF7 is associated with poor prognosis in datasets

**Fig. 1:**
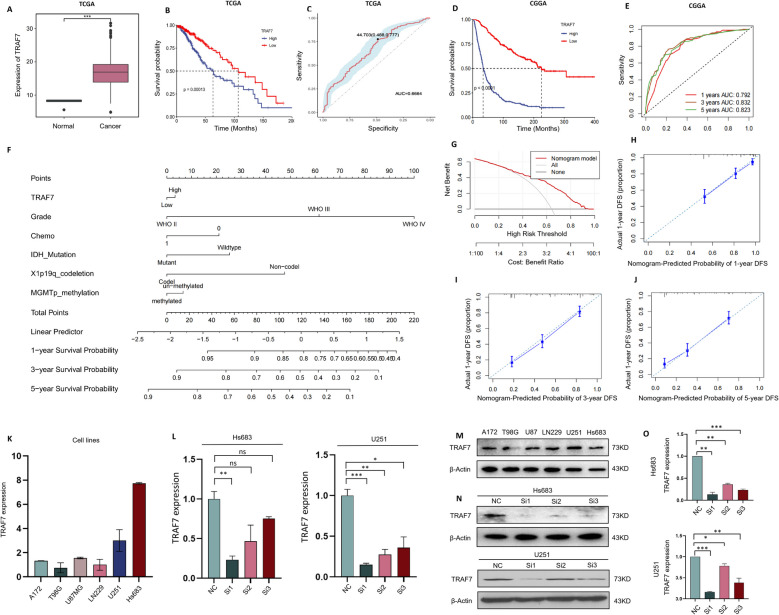
High expression of TRAF7 is closely associated with poor prognosis in glioma. **A** The expression of TRAF7 mRNA was quantitatively analyzed between the glioma tumor site and normal brain tissues based on TCGA database. **B**-**C** Kaplan–Meier survival and ROC curves using TCGA. **D**-**E** Kaplan–Meier survival curves and ROC for predicting the 1-, 3- and 5-year overall survival (*p* < 0.0001) in CCGA. **F** The Nomogram model was built to analyze prognostic factors in 1-, 3-, and 5-year OS of glioma patients. **G** Decision curve analysis (DCA) for the clinical benefits and application of the nomogram. **H**-**J** Calibration curves of the nomogram model for 1-year, 3-year, and 5-year OS. **K** TRAF7 expression were examined by qRT-PCR in six glioma cell lines. **L** The knockdown efficiency of TRAF7 mRNA in Hs683 and U251 cells. **M** The protein expression of TRAF7 was conducted by western blot. **N**–**O** TRAF7 protein expression was quantified in Hs683 and U251 cells. Data are presented as the mean ± SD

High-level expressions of TRAF7 mRNA were shown in human glioma samples in TCGA database, including high-grade gliomas (HGGs, III-IV grade) and low-grade gliomas (LGGs, I-II grade) (Fig. 1A). The top 50 differentially expressed genes (DEGs) were also identified when comparing normal brain tissues to glioma tissues (Fig. S1). As illustrated in Fig. 1B-E, elevated TRAF7 expression was correlated with unfavorable OS in TCGA (*p* = 0.00013) and CGGA database (*p* < 0.0001). Therefore, AUC results suggested that higher TRAF7 expression may act as a potential diagnostic marker in glioma. 1-year, 3-year, and 5-year sensitivity of AUC in CGGA were 0.792, 0.832, and 0.823, respectively.

The model integrating all factors from the multivariate cox regression was displayed as a nomogram in Fig. 1F. This model demonstrated that tumor grade contributed the most to prognosis, followed by TRAF7 expression, chemotherapy, IDH1 mutation, X1p19q codeletion and MGMTp methylation. Besides, the 1-, 3-, and 5-year survival probabilities were also estimated by the nomogram. Decision curve analysis (DCA) supported the nomogram's high clinical utility (Fig. 1G). Calibration curves were also constructed to predict survival probability as shown in Fig. 1H-J. The plots demonstrated strong concordance between the nomogram's forecasts and real-world outcomes for 1-, 3-, and 5-year overall survival. The correlation of TRAF7 expression and IDH1 mutation status, gender, race, and age were also examined in the TCGA database (Fig. S2). We also combined IDH mutation, MGMT methylation, and TRAF7 expression to predict survival and conducted multivariate regression analysis. The results demonstrated that the combination model provided the best predictive effect compared to any single factor alone (Fig. S3).

To explore the expression of TRAF7 in glioma *in vitro*, we determined the TRAF7 protein and mRNA levels in six human glioma cell lines, including A172, T98G, U87, LN229, U251, and Hs683. This set consisted of five GBM cell lines and one LGG cell line. As shown in Fig. 1K, U251 and Hs683 cells were selected for both *in vitro* and *in vivo* experiments, because these cell lines exhibited significant elevation in TRAF7 mRNA expression levels. Subsequently, siRNA1 demonstrated the highest knockdown efficiency at mRNA expression levels in Hs683 and U251 cells (Fig. 1L). We also explored the protein expression of TRAF7 at different glioma cell lines (Fig. 1M-O).

### TRAF7 high level expression is closely related with poor prognosis in glioma patients with clinical characteristics

**Fig. 2:**
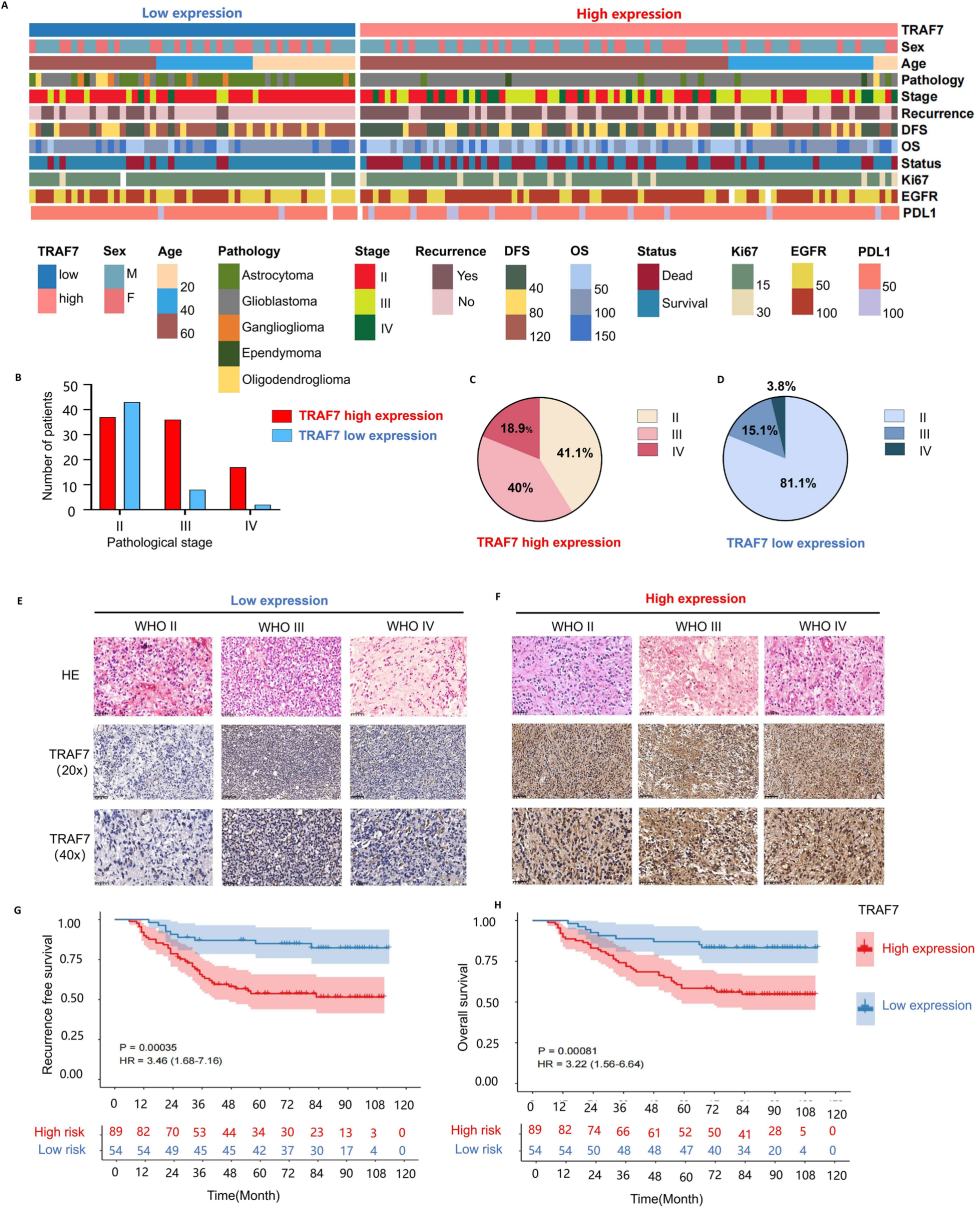
The clinical characteristics of glioma patients. **A** Heat map of 143 glioma patients with clinical characteristics, including 76 recurrent samples. **B**-**D** The TRAF7 expression levels of glioma patients displayed differences in their pathological stages. **E**–**F** The representative images of Hematoxylin and IHC staining were shown in different TRAF7 expression groups (The scale bar for HE staining and 40 × IHC staining is 50 µm, and the scale bar for 20 × IHC staining is 100 µm). **G**-**H** Survival curves of RFS and OS glioma patients with varying levels of TRAF7 expression

The baseline characteristics of 143 glioma patients are presented in Table 1, including 76 recurrent samples. The heat map was shown in Fig. 2A. According to analysis of the heat map, glioma patients with high-grade exhibited stronger relationship with the relative expression of TRAF7 (Fig. 2B-D). The original tissue microarray was stained with HE and TRAF7 IHC in Fig. S4 and Fig. S5, respectively. Statistical analysis also revealed significant associations between high TRAF7 expression and factors such as age (≥ 45 years), WHO grade (III and IV), and clinicopathological subtype (glioblastoma) in Table 1. Utilizing the immunohistochemistry (IHC) scoring system, 37.76% (54/143) of tumors were classified as high TRAF7 protein expression (Fig. 2E, F).

The Kaplan–Meier method was utilized to determine the prognostic impact of TRAF7. Results from the survival analysis showed that individuals with increased TRAF7 levels had a median disease-free survival (DFS) of 47.0 months compared to 85.5 months (95% confidence interval [CI], 1.677–7.155, *p* = 0.001) for those with low TRAF7 expression (Fig. 2G). Similarly, the cohort with higher TRAF7 expression levels showed OS was 77.0 months, while it was 87.0 months (95% CI, 1.562–6.641, *p* = 0.002) in the low TRAF7 expression group (Fig. 2H). Furthermore, through univariate Cox regression analysis in Table 2, TRAF7 expression was validated as an independent prognostic indicator for both OS and DFS in glioma.

**Table 2:** Univariate Cox regression of TRAF7 expression for overall survival in glioma patients

Variable	HR(95% CI)	*p *value
Gender	1.424 (0.729–2.782)	0.301
Age (≥ 45 vs < 45)	1.038 (1.019–1.059)	0.000
Grade (III vs II)	8.759 (3.727–20.586)	0.000
Grade (IV vs II)	68.509 (24.799–189.262)	0.000
DFS	3.464 (1.677–7.155)	0.001
OS	3.220 (1.562–6.641)	0.002
Ki67	3.296 (0.408–26.611)	0.263
EGFR	1.483 (0.725–3.035)	0.280
PDL1	1.610 (0.455–5.695)	0.460
TRAF7	1.021 (1.003–1.039)	0.021

### The cell function of TRAF7 knockdown inhibits proliferation and migration *in vitro*

**Fig. 3:**
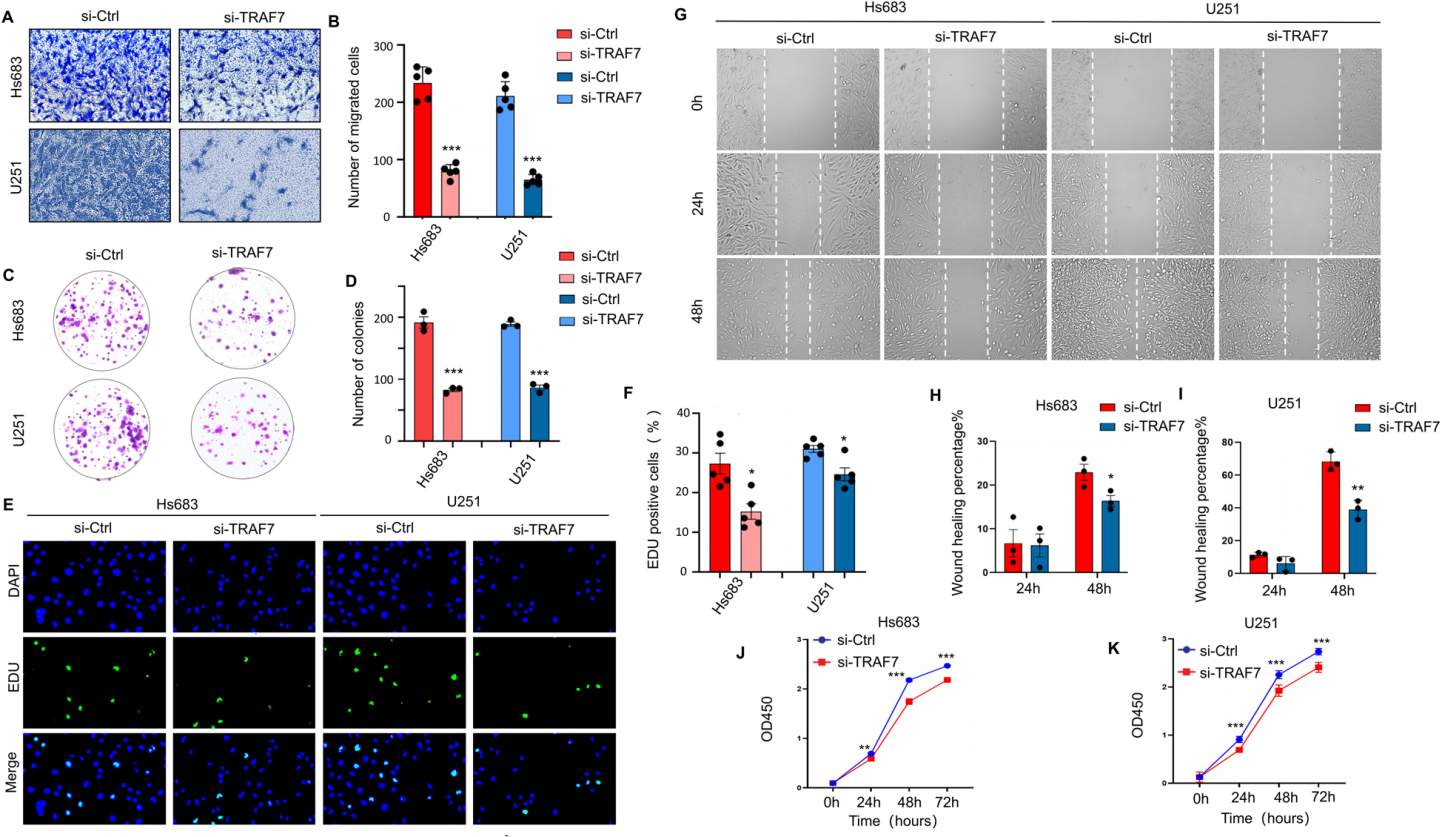
The knockdown of TRAF7 inhibits cell proliferation and migration *in vitro*. **A**-**B** Transwell assays in Hs683 and U251 cells. **C**-**D** Colony formation assays visualized on day 14. **E**–**F** The Hs683 and U251 cell lines with TRAF7 knockdown significantly inhibited cell proliferation by EDU assays. **G**-**I** Wound healing assays and migration rate at 24 and 48 h. **J**-**K** Cell viability was determined by the CCK-8 assay. Data are presented as the mean ± SEM from three independent experiments. *p < 0.05, **p < 0.01, and ***p < 0.001

To investigate the function of TRAF7 on the proliferation and migration in the model of glioma cell line, a series of *in vitro* assays were performed. The transwell assays demonstrated that the siTRAF7 group reduced cell migration by approximately 60% compared to the control group (Fig. 3A, B). Wound-healing assays were also performed to indicate a substantial inhibition of cell migration because of TRAF7 knockdown (Fig. 3G-I). Wound closure in the siTRAF7 group was slightly slower compared to the control group at a 24-h time point. However, the siTRAF7 group exhibited significantly impaired wound closure after 48 h, with a wound healing percentage of approximately 16.4%, compared to 23.0% in the control group in Hs683 cells (*p* < 0.05). Similarly, in U251 cells, the wound healing percentage was 39.0% in the siTRAF7 group versus 68.3% in the control group (*p* < 0.01).

siTRAF7 treatment induced a substantial decrease in colony formation, decreasing the number of colonies by about 50% as opposed to the control group. Specifically, in Hs683 cells, the mean number of colonies was reduced from 178 ± 17 in the control group to 89 ± 5 in the siTRAF7-treated group (*p* < 0.001). The results were consistent with those of previous studies in U251 cell line (Fig. 3C, D). As shown in Fig. 3E-F, the EdU assay results showed a significant inhibition of cell proliferation following TRAF7 knockdown. The amount of EdU-positive cell population decreased from 27.33% in the control group to 15.29% in the siTRAF7 group in Hs683 cells (*p* < 0.05). The similar results were observed in the U251 cell line, further demonstrated a substantial decrease in DNA synthesis following TRAF7 knockdown. Additionally, the CCK8 assay also indicated that the downregulation of TRAF7 led to the cell viability suppression after 48 h (Fig. 3J, K).

The cellular and molecular heterogeneity of gliomas presents significant challenges to conventional treatment strategies [19, 20]. Considering that, for some real-life patients, TRAF7 expression is lower and more transient, and in conjunction with the data from Fig. 1K and M, we performed TRAF7 overexpression in the TRAF7 low-expression cell line T98G. Our results showed that, in T98G cells, colony formation assays and transwell assays demonstrated that the overexpression of TRAF7 significantly enhanced proliferation, migration, and invasion abilities compared to the control group (Fig. S6).

### TRAF7 knockdown induces cellular senescence and cell-cycle arrest

**Fig. 4:**
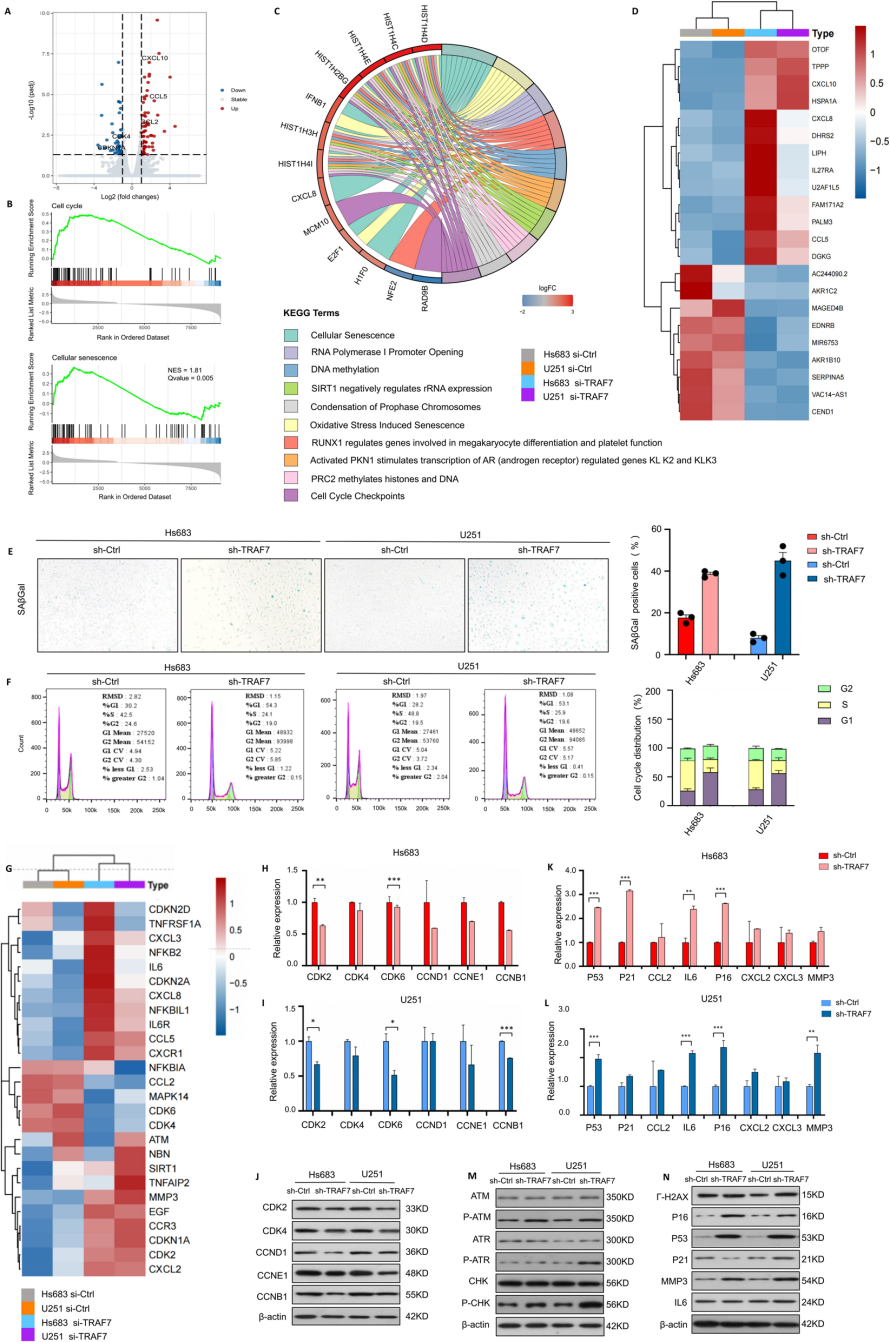
TRAF7 loss sensitizes glioma to senescence and G0/G1 arrest by RNA sequencing. **A** Volcano plot of differentially expressed genes between the control group and siTRAF7 group in the Hs683 and U251 cells. **B** GSEA revealed the enrichment of differentially expressed genes. **C** The pathway enrichment analysis of Kyoto Encyclopedia of Genes and Genomes (KEGG). **D** Heatmap of the differentially expressed (|log2FC|> 2 and *p*-value < 0.05) genes via RNA-seq. **E** The Hs683 and U251 cells with TRAF7 knockdown induced senescence. **F** Knockdown of TRAF7 induced G0/G1 arrest of Hs683 and U251 cells. **G** The heatmap of senescence and cell-cycle related genes. **H**-**I** mRNA expression of G0/G1 arrest related genes. **J** Protein expression of G0/G1 arrest related genes in Hs683 and U251 cells. **K**-**L** mRNA expression of senescence related genes in Hs683 and U251 cells. **M**–**N** The protein expression of G0/G1 arrest and senescence related genes

To further discover the potential mechanism of TRAF7 inhibition, RNA-seq analysis was performed on the siTRAF7 and vector groups. We identified 27,094 transcripts, of which 188 were differentially expressed between the two groups with an average padj < 0.05, log2FoldChange > 1 (Fig. 4A). This included 136 upregulated and 52 downregulated genes in the TRAF7 knockdown samples as compared to controls. Furthermore, Gene set enrichment analysis (GSEA) and Kyoto Encyclopedia of Genes and Genomes (KEGG) analysis were executed and illustrated that cellular senescence and cell cycle were notably activated in the TRAF7 inhibition cell line (Fig. 4B-C). The top 20 differentially expressed genes were presented in Fig. 4D.

To achieve sustained downregulation of TRAF7, shRNAs were stably transfected into U251 and Hs683 cell lines (Fig. S7A-B). We then evaluated the senescence phenotype by a β-galactosidase assay in both cell lines. The results demonstrated that sh-TRAF7 induced a senescence state in cells, compared with the TRAF7 vector group (Fig. 4E). Flow cytometry showed that TRAF7 knockdown could significantly induce G0/G1 arrest in Fig. 4F. Besides, our study also investigated the alteration of key senescence associated genes in the two GBM cell lines. A differential heatmap was generated regarding 26 senescence associated genes by RNA-seq between TRAF7 knockdown and TRAF7 vector group (Fig. 4G).

To further elucidate the mechanisms, we investigated key genes involved in G0/G1 arrest, DNA damage response (DDR) pathways, the p53-p21 axis, and SASP factors at both mRNA and protein levels. As depicted in Fig. 4H-J, compared with the negative control (NC) group, TRAF7 inhibition led to a downregulation of both mRNA and protein levels of essential genes for cell cycle G1/S transition, such as CDK2 (*p* < 0.001), CDK4, CDK6 (*p* < 0.0001), CCND1, CCNE1, and CCNB1 in Hs683 cell lines. Furthermore, sh-TRAF7 induced DNA damage, activating downstream key genes of DDR pathways (γ-H2AX, ATM, ATR, and CHK) and the p53-p21 axis (*p* < 0.0001) at both mRNA and protein levels. The expression of crucial SASP factors, including MMP3, IL-6 (*p* < 0.001), CCL2, CXCL2, and CXCL3, also significant increased in TRAF7 knockdown Hs683 cells (Fig. 4K-N). Similar results were also observed in the U251 cell line.

### Combination of Lomustine (CCNU) and sh-TRAF7 promotes glioma senescence and G0/G1 arrest

**Fig. 5:**
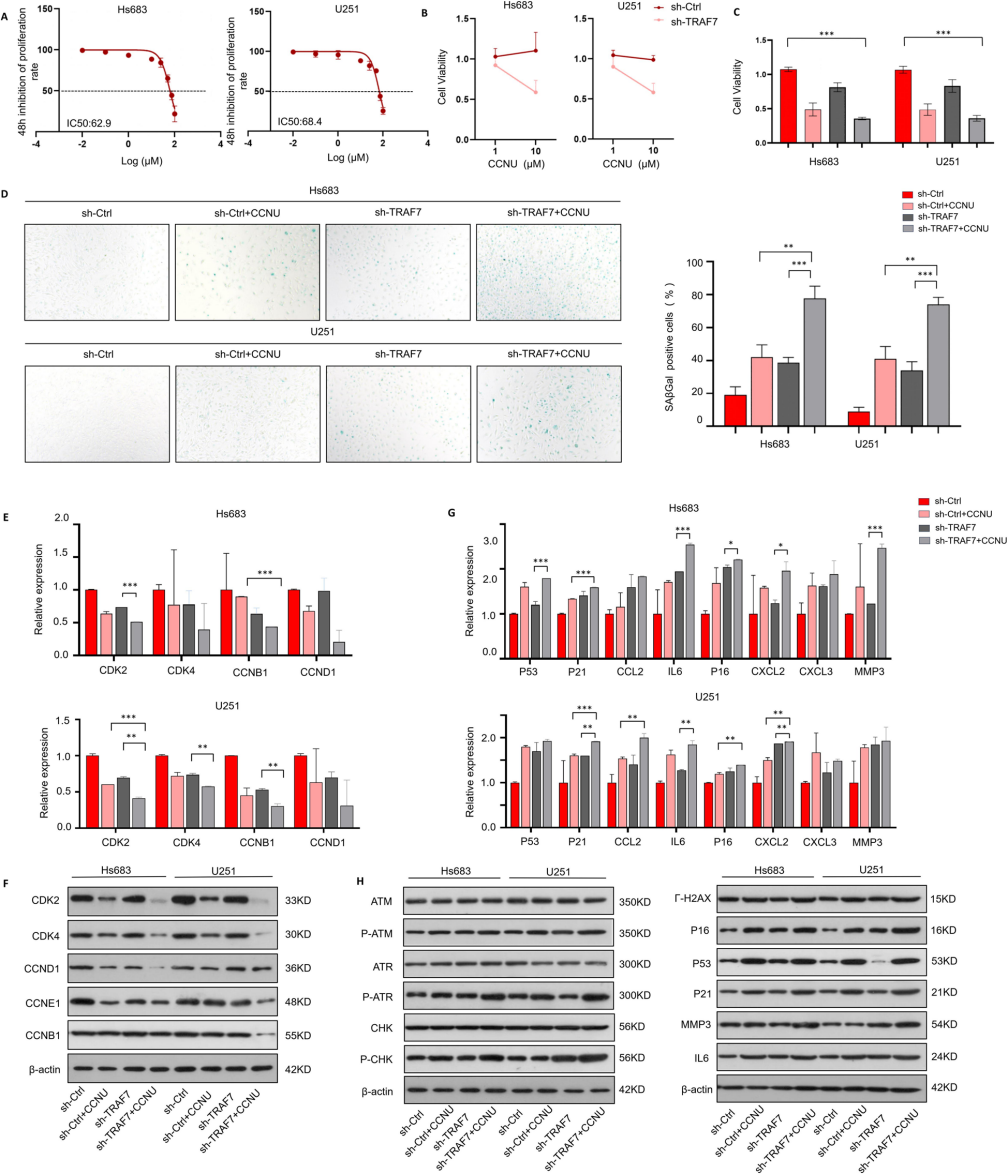
Combination of Lomustine (CCNU) and sh-TRAF7 promotes glioma senescence and G0/G1 arrest. **A** IC50 of lomustine (CCNU). B Cell viability of TRAF7 deficient cell lines treated with low concentrations of lomustine (1 μM and 10 μM) for 12 h. **C** Cell viability of Hs683 and U251 cells treated with lomustine (50 μM). **D** SA-β-gal staining after CCNU treatment of the control group and sh-TRAF7 group in the cells. **E**–**F** The mRNA expression and protein expression of G0/G1 arrest related genes after CCNU treatment. **G**-**H** mRNA expression and protein expression of senescence related genes after CCNU treatment in the cells

Lomustine, palbociclib, and etoposide were recommended as second-line treatments, and all of them were effective in the progression and recurrent gliomas among the CSCO and ASCO guidelines [21–23]. IC50 analysis revealed that lomustine demonstrated the strongest inhibitory effect on proliferation in glioma cell lines (Fig. 5A, Fig. S8). Thus, we chose 50uM lomustine for the subsequent experiment. To further validate the synergistic effect of TRAF7 knockdown and lomustine, cell viability assays were performed 1 μM and 10 μM lomustine as low concentrations. Our results demonstrated that TRAF7-deficient glioma cells exhibited increased sensitivity to lomustine in a dose-dependent manner (Fig. 5B). Moreover, the combination of TRAF7 knockdown and CCNU treatment showed the most potent inhibitory effect, surpassing both the TRAF7 knockdown and lomustine-only treatment groups (Fig. 5C).

Various experiments were conducted to explore whether the effects of combination therapy were associated with senescence. Compared with CCNU or TRAF7 inhibition alone, our results found that the count of senescent cells (measured via ß-galactosidase-senescence assay) was markedly increased to glioma sh-TRAF7 cells treated with CCNU (Fig. 5D). As depicted in Fig. 5E-F and Fig. S9, simultaneous treatment with TRAF7 inhibition and CCNU led to a more pronounced downregulation of crucial genes involved in G0/G1 arrest (CDK2, CDK4, CCND1, CCNE1, and CCNB1) at both mRNA and protein levels. Furthermore, our exploration at mRNA and protein levels revealed that the combination of TRAF7 suppression and CCNU resulted in an enhanced expression of key genes in the DDR pathways, namely γ-H2AX, ATM, ATR, and CHK, as well as the p53-p21 axis. Regarding the examination of key SASP factors (MMP3, IL-6, CCL2, CXCL2, and CXCL3), the combination group exhibited the most significant surge among the four treatment groups (Fig. 5G-H, Fig. S10). Overall, the TRAF7 repression group and lomustine-treated group induced a senescence state, especially in the combination treatment group in two glioma cells.

We further constructed U251 cell line with TRAF7 overexpression (OE-TRAF7). The findings indicated that increased expression of TRAF7 greatly stimulated proliferation and migration, while knockdown of TRAF7 inhibited these functions. Notably, the combination of TRAF7 knockdown and CCNU treatment exhibited the most significant inhibitory effect on cell functions (Fig. S11-15).

### TRAF7 depletion inhibits glioma proliferation and induces cellular senescence through KLF4

**Fig. 6:**
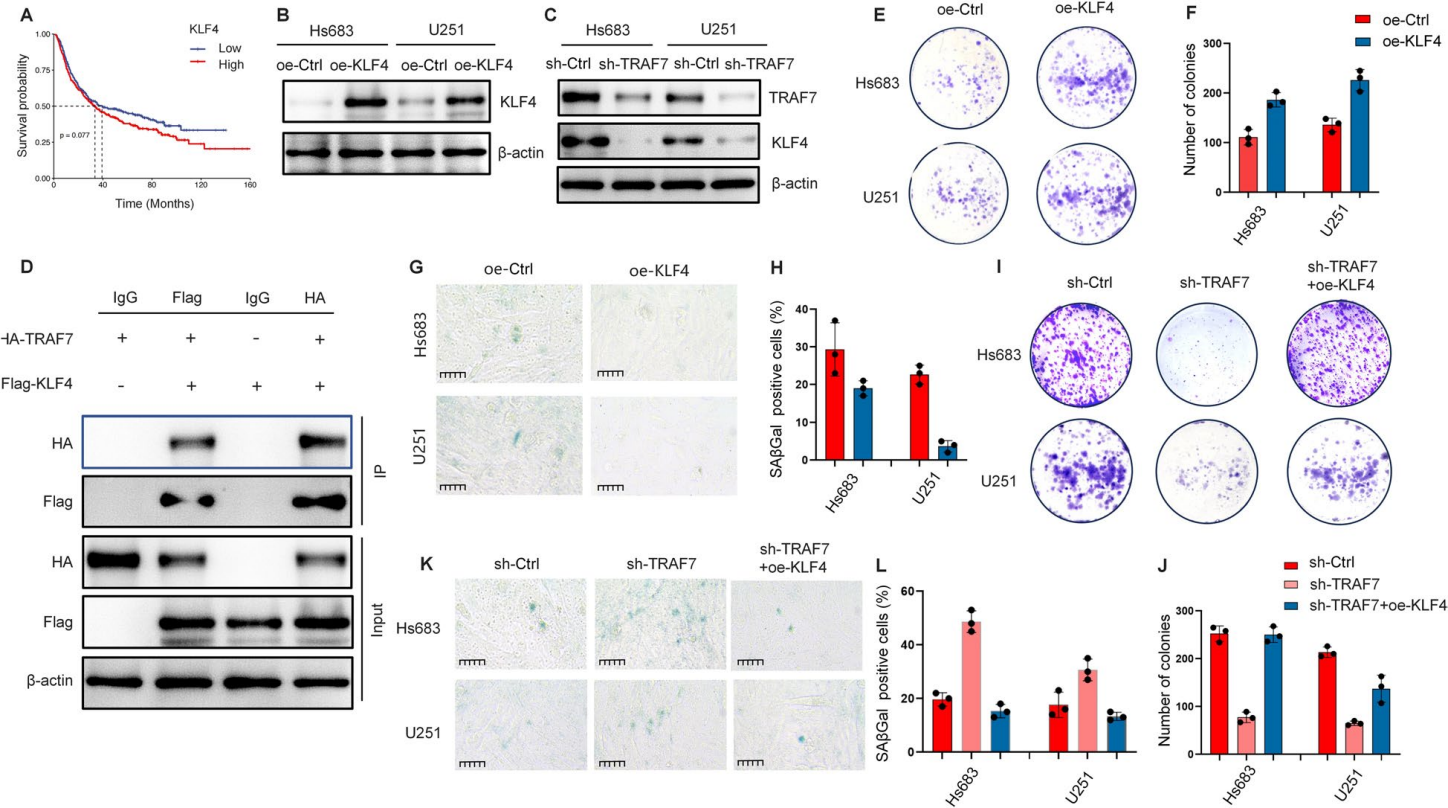
TRAF7 depletion inhibits glioma proliferation and induces senescence via KLF4. **A** Kaplan–Meier survival using TCGA on KLF4. **B** The overexpression of KLF4 in Hs683 and U251 cells. **C** The expression of TRAF7 and KLF4 in the Hs683 and U251 glioma cell lines. **D** Co-immunoprecipitation (Co-IP) assays were performed using lysates from U251 cells transfected with Flag-tagged KLF4 and HA-tagged TRAF7. **E**–**F** Colony formation assays were performed in the glioma cell lines with or without KLF4 overexpression. **G**-**H** SA-β-gal staining was performed in the glioma cell lines (scale bar = 50 µm). **I**-**J** The rescue colony formation assay showed that the inhibitory effects of TRAF7 knockdown on cell proliferation could be rescued by additional KLF4 overexpression in cells. **K**-**L** The rescue SA-β-gal staining assay showed that the promotion effects of TRAF7 knockdown on cell senescence could be rescued by additional KLF4 overexpression in cells (scale bar = 50 µm). *p < 0.05, **p < 0.01, and ***p < 0.001

KLF4, as a component of the OSK genes, plays a crucial role in maintaining stem cell pluripotency. Existing research indicates that OSK-mediated rejuvenation can reverse the aging process [24–26]. A significant interaction between TRAF7 and KLF4 was observed in meningioma [18, 27, 28]. To validate the interaction between TRAF7 and KLF4 in glioma cells, our study found that KLF4 was associated with poor survival in TCGA, and TRAF7 inhibition was accompanied by lower KLF4 expression levels (Fig. 6A-C). Co-IP was performed using lysates from U251 cells co-expressing Flag-tagged KLF4 and HA-tagged TRAF7. As shown in Fig. 6D, KLF4 was enriched in the TRAF7 immunoprecipitates (lane 2), but not in the IgG control (lane 1). This interaction was further confirmed by reciprocal Co-IP, and input lanes confirmed the expression of both proteins.

Then the impact of KLF4 overexpression on glioma cell proliferation and senescence was assessed in the Hs683 and U251 cell lines. Colony formation and SA-β-gal staining assays revealed that KLF4 overexpression significantly enhanced cell proliferation, while reducing the extent of cellular senescence in glioma cells (Fig. 6E-H). Additionally, rescue assays demonstrated that the inhibitory effect of TRAF7 depletion on proliferation, as well as its promotion of cellular aging, could be at least partially reversed by KLF4 overexpression (Fig. 6I-L). These findings suggest that TRAF7 regulates cell proliferation and senescence in a KLF4-dependent manner.

### Development of patient-derived primary and recurrent glioma stem cell (GSCs)

**Fig. 7:**
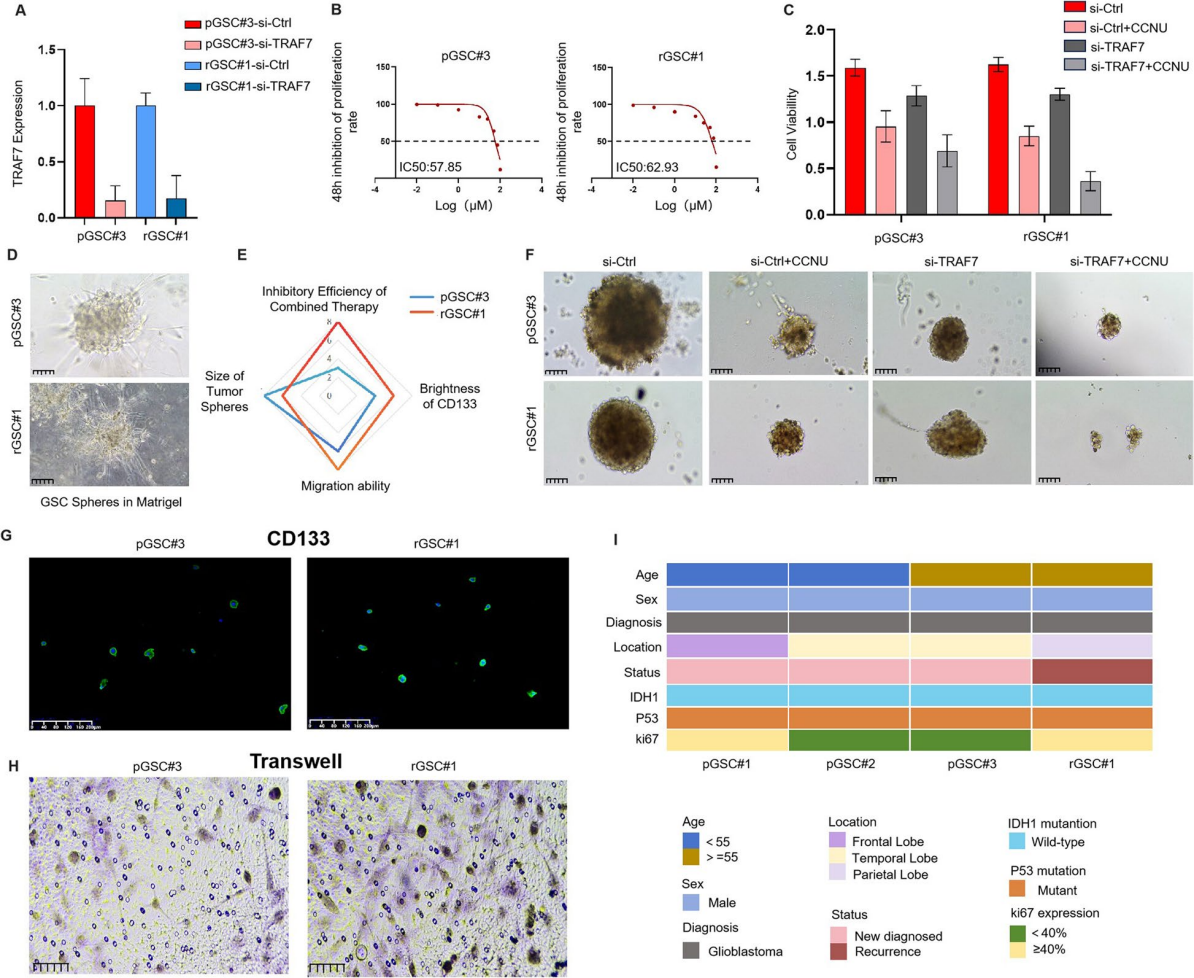
Establishment of patient-derived primary and recurrent glioma stem cell spheres (GSCs). **A** The expression of TRAF7 in primary and recurrent patient-derived glioma cells with or without knockdown. **B** IC50 of Lomustine (CCNU) in primary and recurrent glioma stem cells. **C** Cell viability of primary and recurrent patient-derived glioma cells treated with lomustine (50 μM). **D** Brightfield image of GSC spheres in Matrigel (scale bar = 100 µm). **E** The radar chart provides a comprehensive comparison of different cells, including the inhibitory efficiency of combined therapy, tumor sphere size, CD133 expression levels, and migration capacity. **F** Evaluation of the GSCs formation ability between the control group and si-TRAF7 group treated with or without CCNU (scale bar = 100 µm). **G** The primary and recurrent glioma stem cells were stained with stem cell markers (CD133) (scale bar = 100 µm). **H** Transwell assays of pGSC#3 and rGSC#1 (scale bar = 50 µm). **I** Heat map of three primary glioma patients and one recurrent glioma patient with clinical characteristics

To further validate our conclusions in clinical patients, four glioma tumor tissues were collected, including three primary (pGSC#) and one recurrent (rGSC#) cases with their clinical characteristics (Fig. 7I). TRAF7 knockdown and lomustine treatment were performed on the GSCs (Fig. 7A-B). As shown in Fig. 7C, the combination of TRAF7 suppression and CCNU treatment resulted in the most significant inhibition of proliferation, especially in recurrent patients. The cell viability was 69.2% in the primary patient versus 36.4% in the recurrent patient. Glioma stem cells (GSCs) were cultured in serum-free medium. When mixed with Matrigel, long protrusions resembling neurosphere-like structures were observed, while under low-adherence conditions, GSCs were able to form stable spheres (Fig. 7D). Compared to pGSC#3, the recurrent sample rGSC#1 expressed higher CD133 with stronger characteristics of stemness (Fig. 7G). Transwell assays showed that GSCs derived from recurrent patients exhibited stronger migration ability (Fig. 7H). Sphere formation assays also demonstrated that the combined treatment had a greater effect on sphere formation in GSCs derived from recurrent patients compared to primary patients (Fig. 7F).

Based on the above research results, we created radar charts to qualitatively compare the capabilities of patient-derived primary and recurrent glioma stem cells as follows: the inhibitory efficiency of TRAF7 inhibition and CCNU treatment, tumor sphere size, CD133 expression levels, and migration capacity. It was found that recurrent patients showed stronger sensitivity to combined treatment, higher CD133 expression levels, and enhanced migration capacity compared to primary patients (Fig. 7E).

### Combination of lomustine with sh-TRAF7 suppresses glioma *in vivo*

**Fig. 8:**
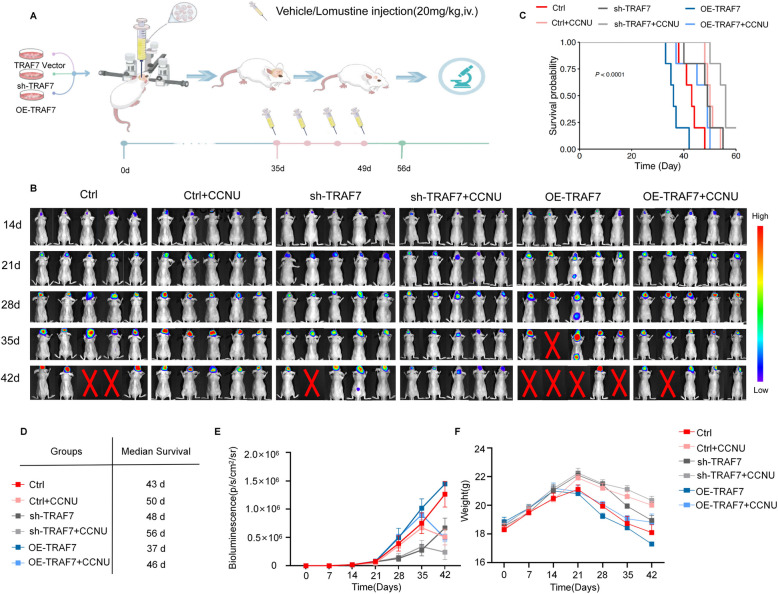
Sh-TRAF7 and Lomustine (CCNU) synergistically inhibit glioma tumor growth*.* **A** Schematic illustration of the experimental design for the glioma orthotopic implantation model, and evaluation of brain tumor growth after injection CCNU. **B** Bioluminescence images of nude mice bearing glioma orthotopic xenograft (*n* = 5) were shown on days 14, 21, 28, 35, and 42. The nude mice were inoculated with U251 cells transfected with luciferase, divided into six groups: (1) Control, (2) Control + CCNU, (3) sh-TRAF7, (4) sh-TRAF7 + CCNU, (5) OE-TRAF7, (6) OE-TRAF7 + CCNU. The crossing in the blank area indicated that the corresponding mouse had died. **C**-**D** Survival curves of glioma-bearing mice, with median survival times listed in the accompanying table. **E** Quantitative analysis of bioluminescence. **F** The curves of body weight in nude mice

To evaluate the regulatory roles of TRAF7, the efficiency of OE-TRAF7 in the U251 cell line was confirmed by western blotting (Fig. S7C). *In vivo* experiments, the U251 cell line consisted of TRAF7 control, sh-TRAF7, and OE-TRAF7 cell lines. Nude mice bearing U251 cell line glioma orthotopic xenograft (*n* = 5) were divided into six groups: (1) Control, (2) Control + CCNU, (3) sh-TRAF7, (4) sh-TRAF7 + CCNU, (5) OE-TRAF7, (6) OE-TRAF7 + CCNU. Four weeks after intracranially injection glioma cell lines, mice were treated with medicine (CCNU 20 mg/kg) twice a week (Fig. 8A). Bioluminescence images of nude mice bearing glioma orthotopic xenograft (*n* = 5) were shown on days 14, 21, 28, 35, and 42 (Fig. 8B). The crossing in the blank area indicated that the corresponding mouse had died. The group with sh-TRAF7 or CCNU treatment displayed a substantial reduction of the tumor growth as opposed to the control group. Fluorescence quantification was depicted in Fig. 8E. The median survival curves for the six groups were presented in Fig. 8C-D, with detailed median survival times provided in the accompanying table. In the combination of sh-TRAF7 plus CCNU group, one of the five longest-surviving mice had a survival period of 64 days, and the median survival time in this group was significantly longer than the other groups. As shown in Fig. 8F, there were no significant weight differences among the six groups of mice. Systemic toxicity evaluation, as shown in Fig. S16, revealed no apparent toxicity in any treatment group. Blood samples taken after four cycles of CCNU treatment were analyzed for blood chemistry and liver/kidney function (Fig. S17-18). The results showed that the levels of white blood cells (including neutrophils, lymphocytes, and monocytes) and platelets were slightly lower in the CCNU-treated groups compared to the untreated groups. These findings suggest that upregulated TRAF7 expression is connected with worse prognosis, indicating that TRAF7 may function as both a biomarker for prognosis and a therapeutic target.

### Histological analyses of lomustine (CCNU) and sh-TRAF7 via mediating cellular senescence and cell-cycle arrest *in vivo*

**Fig. 9:**
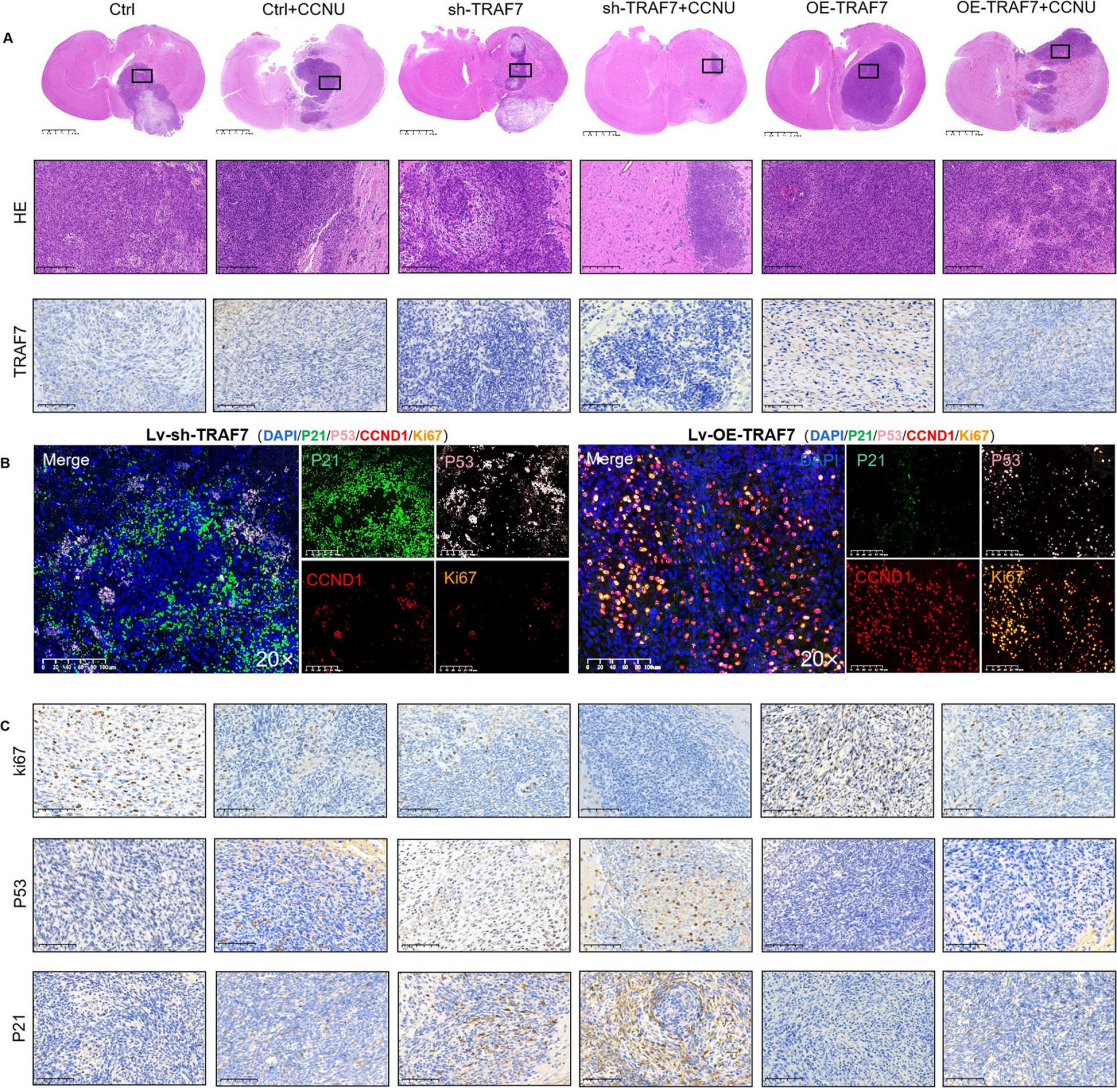
Histological analyses of TRAF7 inhibition and lomustine via mediating cellular senescence and G0/G1 arrest *in vivo.* **A** The whole brain of glioma nude mice were stained HE and TRAF7 (Full scan: scale bar = 2.5 mm; higher-magnification part below: scale bar = 100 µm). **B** Multiplex immunofluorescence staining image of cellular senescence markers (P21 and P53) and G0/G1 arrest markers (CCND1) and Ki67 in the tumor tissue. **C** IHC staining the cellular senescence markers (P21 and P53) and Ki67 in tumor tissue sections (scale bar = 100 µm)

The whole brain of glioma nude mice was collected for HE staining, immunohistochemical staining, and multiplex immunofluorescence. The HE sections clearly showed that the spatial distribution of tumors in brain tissue (Fig. 9A). As shown in the HE whole-brain sections, the brain tumor tissue in the sh-TRAF7 group exhibited higher levels of cavitation and fibrosis. Compared to the untreated group, the tumor tissue became more dispersed and fragmented following treatment with CCNU. This result indicated that the sh-TRAF7 groups and the CCNU-treated group suppressed tumor proliferation, while the OE-TRAF7 group boosted tumor proliferation. The combination of sh-TRAF7 and CCNU treatment was more efficacious than conventional CCNU chemotherapy. The TRAF7 expression of glioma whole-brain sections was consistent with the *in vitro* results.

**Fig. 10:**
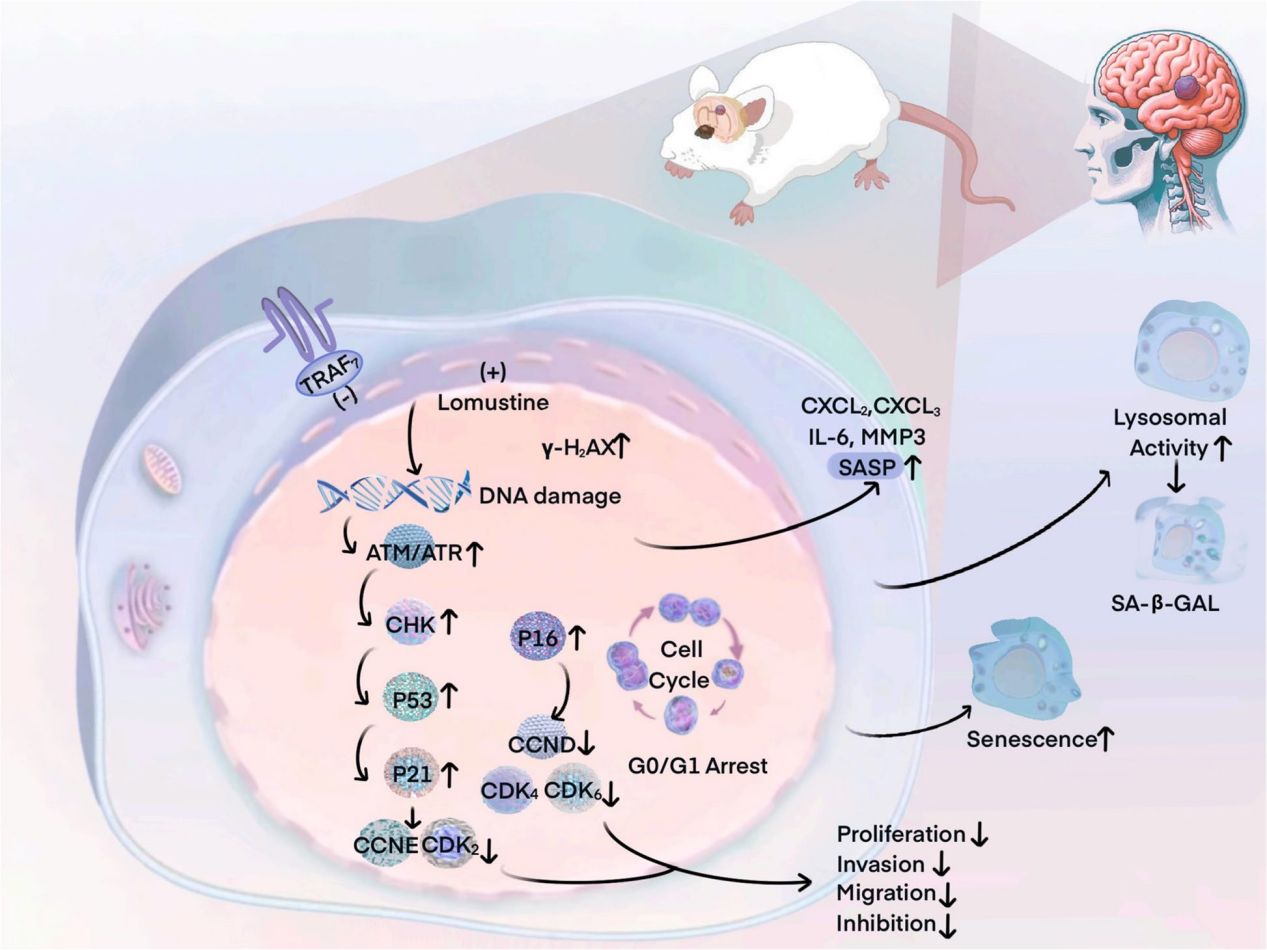
A schematic mechanism overview of sh-TRAF7 and lomustine via mediating cellular senescence and G0/G1 arrest synergistic inhibit glioma tumor growth

Our study further conducted the representative protein markers of cellular senescence markers (P21 and P53) and G0/G1 arrest markers (CCND1, CDK2) in whole-brain sections via multiplex immunofluorescence staining and immunohistochemistry (Fig. 9B-C, Fig. S19). We found that TRAF7 inhibition induced cellular senescence, promoted G0/G1 arrest, and suppressed tumor proliferation (Fig. 10).

## Discussion

Our results showed that TRAF7 restraint and lomustine (CCNU) induced a senescence-state to inhibit the progression and recurrence in glioma. Unfortunately, the current therapeutic methods for the progression and recurrence of glioma remain limited [29, 30]. CCNU, a conventional alkylating agent, is considered as one of the primary choices for recurrent glioma following the failure of first-line treatments according to the NCCN guidelines [31–34]. CCNU only served as a control arm for various other chemotherapeutic agents in the management of recurrent glioma following initial treatments with TMZ or alternative chemotherapeutic agents [23, 35–37]. However, none of the studies exhibited significant superiority over CCNU in terms of efficacy. Our findings suggest that CCNU is highly effective on the patients with high TRAF7 expression. This highlights the significance of CCNU, especially for the recurrent glioma patients with specific gene mutations that should be further investigated.

The function of TRAF7 mutation has been limited reported in brain tumors, and few studies pay attention to selecting therapeutic medicine depending on the TRAF7 expression in the management of glioma. Researches on TRAF7 and its associated tumor signal transduction have recently received significant attention. TRAF7 was considered as a negative regulator which is closely related to poor prognosis, through degradting TBK1, inhibiting IRF3 production and IFN-β activation, to suppress innate immune signaling [38]. Mo Zhang et al. reported that TRAF7 targeted the HOXA5/SPRY2–MEK/ERK signaling axis, contributing to the progression of prostate cancer [39]. A similar regulatory effect was also found in adenomatoid tumor and myeloid leukemia [40, 41].

Herein, we identified that TRAF7 interacts with KLF4 to exert its oncogenic functions. Numerous studies have reported that TRAF7 mutations are frequently found alongside KLF4 mutations in meningiomas [28, 48]. Additionally, other research has shown that TRAF7 influences KLF4 by interacting with its N-terminus and promoting its degradation through ubiquitination in hepatocellular carcinoma (HCC) [49]. Our rescue assays further confirmed that TRAF7 promotes glioma progression in a KLF4-dependent manner. These findings suggest that targeting the TRAF7/KLF4 axis could be a promising therapeutic strategy for glioma. Besides, there are many other proteins that may interact with TRAF7 as shown in Fig. 4D. For example, RNA-seq analysis revealed a significant upregulation of HSPA1A (Hsp70-1) following TRAF7 inhibition. Hsp70, a heat shock protein (HSP) linked to cancer prognosis, plays a crucial role in preventing the denaturation or unfolding of cellular proteins in response to stress or elevated temperatures [42–46]. Evidence indicates that extracellular Hsp70 is capable of stimulating both innate and adaptive immune responses against tumors [47].

The association between TRAF7 suppression and CCNU sensitivity has never been reported. In the present study, we identified CCNU as a clinically applicable drug that synergizes with TRAF7 inhibitors in glioma through promoting a senescence-state. We found that downregulation of TRAF7 expression inhibited glioma progression and recurrence, as validated by the TCGA, CGGA datasets, and the tumor tissue of glioma patients. RNA seq demonstrated that senescence-state was significantly activated after TRAF7 inhibition.

CCNU is well known to exert the accumulation of DNA adducts and activation of p53-p21^CIP1^ axis during senescence [50]. CCNU demonstrated anti-proliferative and senescent effects in the organoid culture of therapeutic-resistant glioma cells [51]. Senescence is linked to a persistent DNA damage response (DDR) state, characterized by elevated deposition of γ-H2Ax (phosphorylated histone H2AX at Ser139) and activation of a kinase cascade involving ATM/ATR/CHK. This cascade ultimately results in the activation of the p53-p21 axis [52, 53]. Besides, senescent cells can also influence their tumor microenvironment by producing a senescence-associated secretory phenotype (SASP) [54, 55]. Previous studies have found that SASP inhibits tumorigenesis by reinforcing the senescence growth arrest *in vitro* through an autocrine positive-feedback loop and inducing nonmalignant proliferating neighbor cells to undergo senescence [56–58]. In our study, TRAF7 suppression combined with CCNU treatment had a synergistic antitumor effect both *in vivo*, *in vitro *and in patient-derived GSC assays through the DDR pathways, p53-p21 axis and the regulation of SASP factors.

The absence of TRAF7 promoted a senescence state and altered the expression of key genes involved in the SASP (CCL2, IL-6, CXCL2, MMP3, etc.). SASP secreted by senescent cells can lead to the accumulation of various senescence-associated pathological features, thereby further promoting tumor progression. For example, Stella Victorelli et al. reported that mitochondrial outer membrane permeabilization (MOMP) in a subset of mitochondria, termed minority MOMP (miMOMP), drives the SASP through the cGAS-STING pathway [59]. Rutin, studied as a targeted agent for SASP, targets senescent cells by dampening SASP expression and restraining the acute stress-associated phenotype (ASAP) through interference with ATM interactions, thereby improving chemotherapy efficiency [60]. However, contrary to previous studies indicating that the combination of MAPK and CDK4/6 inhibitors induces RB protein-mediated cellular senescence and SASP activation, thereby enhancing NK cell immune surveillance and leading to the death of KRAS-mutant lung cancer cells [61]. Our research also found that the absence of TRAF7 expression inhibited glioma growth while promoting SASP expression, confirmed by the *in vitro* experiments. This discrepancy may be due to the nature of the inducing stressor leading to the heterogeneous composition of the SASP at different cell type and senescence stage, making its effects difficult to predict [62, 63]. Besides, SASP may be beneficial by promoting tumor-suppressive paracrine senescence in the tumor microenvironment and reminding the immune system to clear senescent cells [64].

Most of our data were analyzed from public datasets and mice models bearing glioma orthotopic xenograft. Compared to the untreated group, it is gratifying to see that mice treated with the CCNU did not lose any weight, the liver and kidney function tests showed no significant abnormalities. Blood samples obtained from mice after four cycles of concurrent chemotherapy showed slight bone marrow suppression. Thus, inhibition of TRAF7 checkpoint may performed a promising therapeutic strategy and CCNU markedly enhances treatment response in high-expression TRAF7 subtype of glioma. Our findings could represent a significant step toward enhancing the understanding of TRAF7 signaling, which may be crucial for developing more precise therapeutic strategies in the progression and recurrence of glioma.

## Conclusion

TRAF7 has the potential to be a prognostic indicator and a prospective therapeutic target in glioma patients. Our study suggested that the second-line lomustine regulating cellular senescence could be recommended as the optimal choice in the progression and recurrence glioma patients with high-level TRAF7 expression.

## Data Availability

All data in this study are available upon request.

## Abbreviations

ASCO: American Society of Clinical Oncology
CCNU: Lomustine
CGGA: Chinese Glioma Genome Atlas
Co-IP: Co-immunoprecipitation
CSCO: Chinese Society of Clinical Oncology
DCA: Decision Curve Analysis
DDR: DNA Damage Response
GBM: Glioblastoma
GSC: Glioma Stem Cell
GSEA: Gene Set Enrichment Analysis
HCC: Hepatocellular Carcinoma
HGG: High-Grade Glioma
IC50: Half Maximal Inhibitory Concentration
IHC: Immunohistochemistry
IRF: Interferon-Regulatory Factor
KEGG: Kyoto Encyclopedia of Genes and Genomes
KLF4: Krüppel-Like Factor 4
LGG: Low-Grade Glioma
MOMP: Mitochondrial Outer Membrane Permeabilization
NCCN: National Comprehensive Cancer Network
NF-κBs: Nuclear Factor-κBs
OS: Overall Survival
RNA-seq: RNA Sequencing
RT-qPCR: Real-Time Quantitative PCR
SASP: Senescence-Associated Secretory Phenotype
TCGA: The Cancer Genome Atlas
TRAF7: TNF Receptor-Associated Factor 7
γ-H2Ax: Phosphorylated Histone H2AX at Ser139
